# Progressive stenosis and radiological findings of vasculitis over the entire internal carotid artery in moyamoya vasculopathy associated with graves’ disease: a case report and review of the literature

**DOI:** 10.1186/s12883-019-1262-1

**Published:** 2019-03-02

**Authors:** Hiroto Ito, Syunsuke Yokoi, Kinya Yokoyama, Takumi Asai, Kenji Uda, Yoshio Araki, Syuntaro Takasu, Rei Kobayashi, Hisashi Okada, Satoshi Okuda

**Affiliations:** 1 Department of Neurology, National Hospital Organization Nagoya Medical Center, 4-1-1 Sannomaru, Naka-ku, Nagoya, Aichi Japan; 2 Department of Rheumatology, National Hospital Organization Nagoya Medical Center, 4-1-1 Sannomaru, Naka-ku, Nagoya, Aichi Japan; 3 Department of Neurosurgery, National Hospital Organization Nagoya Medical Center, 4-1-1 Sannomaru, Naka-ku, Nagoya, Aichi Japan; 4 Department of Neurosurgery, Nagoya University Graduate School of Medicine, Tsurumai-cho 65, Showa-ku, Nagoya, Aichi Japan; 5 Department of Neurosurgery, Japanese Red Cross Nagoya Daini Hospital, 2-9 Myouken-cho, Showa-ku, Nagoya, Aichi Japan

**Keywords:** Moyamoya vasculopathy, Graves’ disease, Recurrence, Champagne bottle neck sign, Vasculitis

## Abstract

**Background:**

Moyamoya vasculopathy (MMV) associated with Graves’ disease (GD) is a rare condition resulting in ischemic stroke accompanied by thyrotoxicity. Radiological findings of vasculitis have been reported in the walls of distal internal carotid arteries (ICAs) in these patients; however, no reports have described in detail the processes of progression of the lesions in the proximal ICA. Moreover, treatments to prevent recurrence of ischemic stroke and progression of MMV have not yet been sufficiently elucidated.

**Case presentation:**

We report a progressive case of MMV associated with GD and review the literature to clarify relationships among recurrence, progression, thyrotoxicity and treatment. Our patient developed cerebral infarction during thyrotoxicity with no obvious stenosis of ICAs. Five months later, transient ischemic attacks recurred with thyrotoxicity. Antiplatelet therapy and intravenous methylprednisolone stopped the attacks. Stenosis of the left ICA from the proximal to distal portion and champagne bottle neck sign (CBN) were found. She declined any surgery. Afterward, gradual progression with mild thyrotoxicity was observed. Eventually, we found smooth, circumferential, concentric wall thickening with diffuse gadolinium enhancement of the left ICA from the proximal to the distal portion on T1-weighted imaging, suggesting vasculitis radiologically. The clinical and radiological similarities to Takayasu arteritis encouraged us to provide treatment as for vasculitis of medium-to-large vessels. In a euthyroid state and after administration of prednisolone and methotrexate, improved flow in the cerebrovascular arteries on magnetic resonance angiography was observed. Based on our review of the literature, all cases with recurrence or progression were treated with anti-thyroid medication (ATM) alone and accompanied by thyrotoxicity. CBN was observed in all previous cases for which images of the proximal ICA were available.

**Conclusions:**

We report the details of progressive stenosis from a very early stage and radiological findings of vasculitis over the entire ICA in MMV associated with GD. Cerebral infarction can occur with no obvious stenosis of the ICA. We treated the patient as per vasculitis of a medium-to-large vessel. Management of GD by ATM alone seems risky in terms of recurrence. Adequate management of GD and possible vasculitis may be important for preventing recurrence and progression.

**Electronic supplementary material:**

The online version of this article (10.1186/s12883-019-1262-1) contains supplementary material, which is available to authorized users.

## Background

Graves’ disease (GD) is rarely complicated by moyamoya vasculopathy (MMV), resulting in ischemic stroke during thyrotoxicity [1, 2]. Management of GD is considered important to prevent recurrence [3], but MMV may progress despite the control of GD [4]. A case was recently reported in which the wall of the distal ICA was enhanced on contrast-enhanced (CE) T1-weighted imaging (T1WI), suggesting vasculitis [5]. However, no reports have described the details of the processes of progression and the lesions of the proximal ICA in MMV associated with GD. Moreover, results have not been described in these patients after treatment for vasculitis of medium-to-large vessels and several issues remain uncertain, such as treatments to prevent recurrence and progression, and characteristic radiological findings.

## Case presentation

**Fig. 1:**
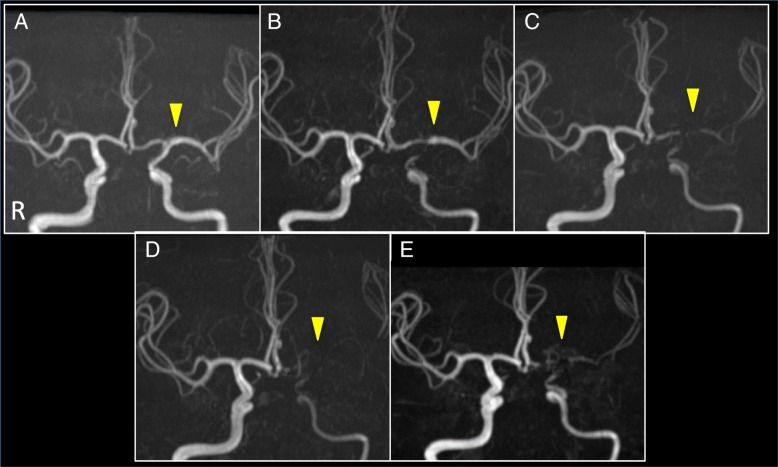
Brain magnetic resonance angiography in the course. **a** Brain MRA did not show obvious stenosis of ICAs. **b**-**d** Stenosis in cerebral arteries on brain MRA progressed during thyrotoxicity. **e** Eighteen months after recurrence, brain MRA suggests improved blood flow through improved flow of the left ICA and MCA

**Fig. 2:**
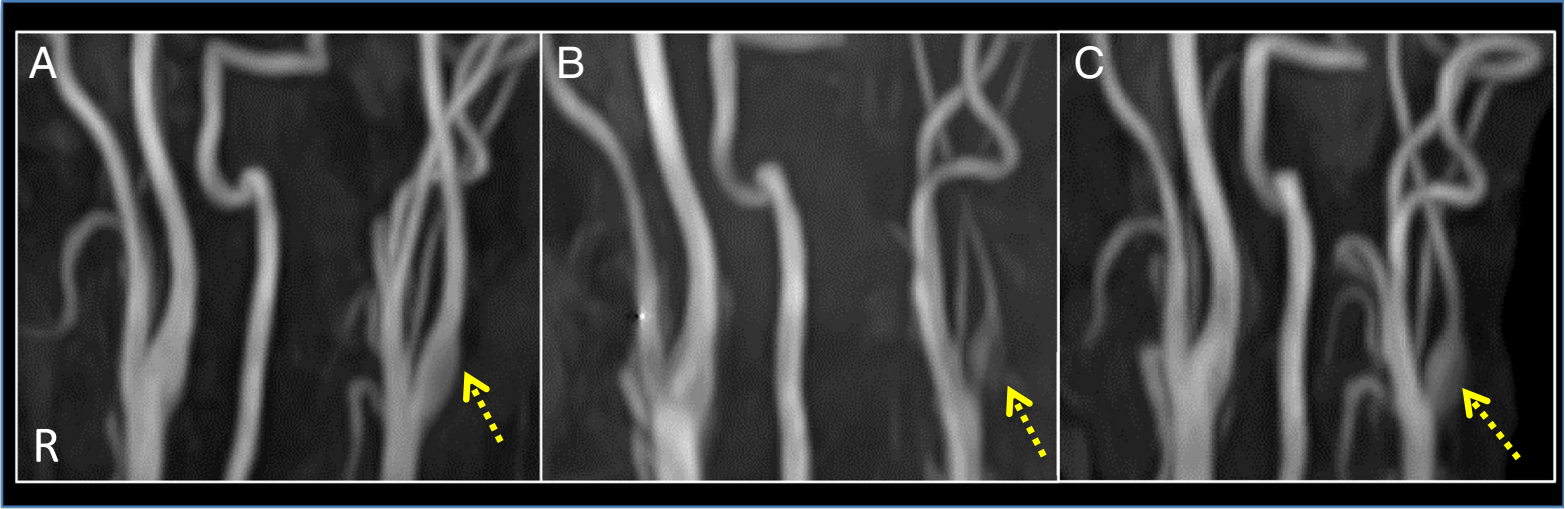
Cervical magnetic resonance angiography in the course. **a** Cervical MRA showed very mild or no obvious stenosis in the first episode. **b** Cervical MRA showed progressed stenosis of the left ICA at proximal portion and CBN was observed in the second episode for the first time. **c** Eighteen months after recurrence, cervical MRA also suggests improved blood flow through improved flow in the proximal left ICA

**Fig. 3:**
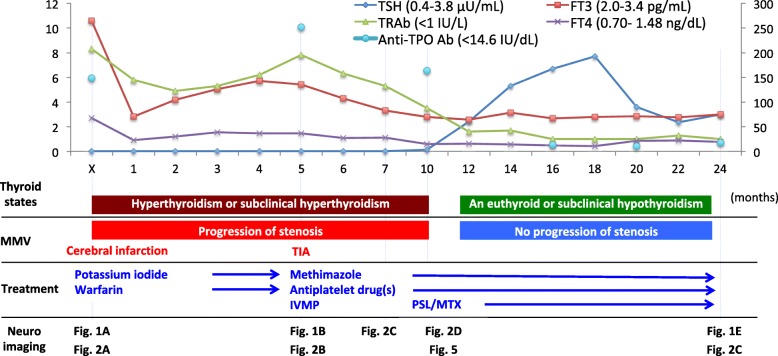
Clinical course of moyamoya vasculopathy and Graves’ disease. Thyroid function and titers of the autoantibodies related to GD (anti-TPO Ab and TRAb) seemed to be associated with progression of MMV and occurrence of ischemic stroke. Thyroid function, anti-TPO Ab and TRAb normalized after administration of PSL, MTX, and in a euthyroid state

A 37-year-old woman presented with gradually progressing weakness of the right arm. She had a medical history of asthma only in her childhood and no notable family history. On physical examination, she showed mild paralysis of the right arm. Although she felt palpitation and sweating at times, exophthalmic and enlarged thyroid lobes were not observed. Diffusion-weighted imaging (DWI) showed cortical and subcortical infarcts in the left MCA territory (Additional file 1: Figure S1), but magnetic resonance angiography (MRA) showed almost-normal cerebral arteries or very mild stenosis of the left ICA (Figs. 1a, 2a). The vessel wall seemed thicker in the left ICA than in the right on three-dimensional (3D)-T1WI, but the difference was not clear (Additional file 2: Figure S2A). Hyperthyroidism [levels of free T3, free T4, and thyroid stimulating hormone (TSH); 10.58 pg/mL, 2.70 ng/dL, and 0.01 μU/mL, respectively], and autoantibodies related to GD [anti-thyroid peroxidase antibody (anti-TPO Ab, 148.0 IU/mL), and TSH receptor antibody (TRAb, 8.3 IU/mL)] were identified (Fig. 3). Other laboratory investigations showed unremarkable results except for leukopenia, anti-SS-A antibody (89.5 IU/mL; normal range, < 7.0 IU/mL), anti-SS-B antibody (12.4 IU/mL; normal range, < 7.0 IU/mL) and thrombin-antithrombin complex (TAT, 2.5 ng/mL; normal range, < 0.3 ng/mL). C-reactive protein (CRP) and erythrocyte sedimentation rate (ESR) were negative. She was diagnosed with GD, but not with Sjogren’s syndrome, based on the normal results of Schirmer’s test and a salivary flow-rate test. Rather than methimazole, she was treated with potassium iodide (150 mg/day) for GD due to leukopenia. MRA excludes over 50% stenosis of intracranial and extracranial cerebral arteries and we could not find any major risks of cardioembolic source of embolism, through electrocardiography, echocardiography, and cardiac rhythm monitoring for over 24 h, so heparin, then warfarin (4 mg/day) were administrated as treatments for stroke as unknown etiology. Mild weakness remained at discharge.

**Fig. 4:**
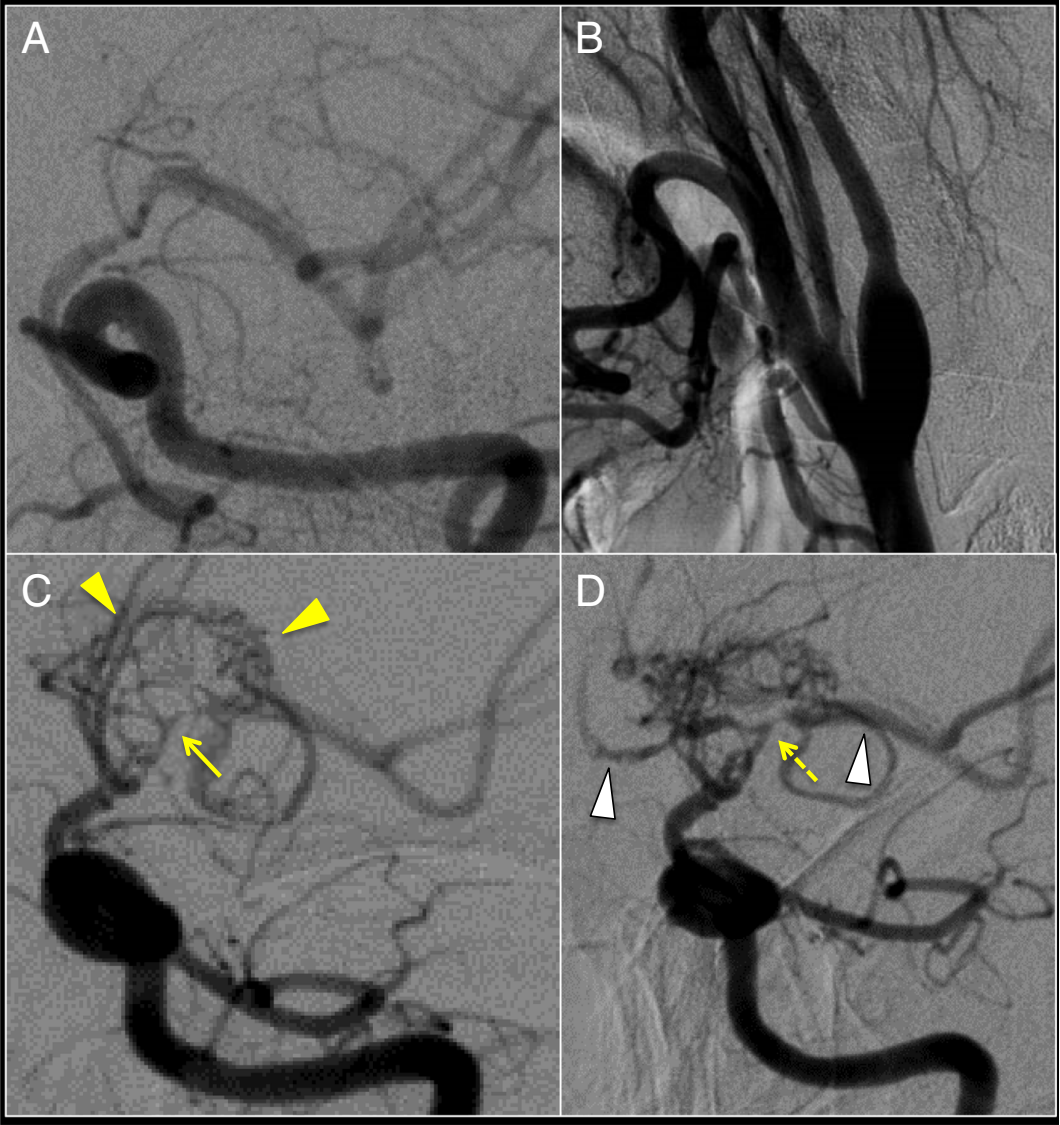
Cerebral angiograms in the second episode, 1 year and 2 years later. **a** Cerebral angiograms of the terminal portion of the left ICA in the second episode showed severe stenosis of the distal portion. **b** Cerebral angiograms of the proximal left ICA showed stenosis at proximal ICA and CBN in the second episode. **c** Cerebral angiograms 12 months after the second episode showed progression of stenosis of the distal portion and net-like vessels might have been developed (arrowhead-yellow), but incomplete occlusion (arrow). **d** Cerebral angiograms 24 months after the second episode showed improved blood flow in the left ACA, MCA and ICA (arrowhead-white), as well as mild improvement of stenosis of the terminal portion of the left ICA (dashed arrow). We consider that since the progression of MMV stopped after administration of PSL and MTX, and in the euthyroid state, cerebral angiograms did not show complete occlusion of distal ICA. Afterward, the net-like vessels might have developed only in the limited space around the distal ICA region. Improved blood flow through the net-like vessels and mild improvement of stenosis in cerebral arteries on cerebral angiograms might have increased blood flow in the left ICA and MCA on MRA

Five months later, she presented with intermittent transient weakness of the right arm and leg. DWI did not show new infarction. Stenosis of the left ICA progressed on MRA (Figs. 1b, 2b). Thyrotoxicity was exacerbated (Fig. 3). Although argatroban was administered, attacks recurred 5 times. We started intravenous methylprednisolone (IVMP; 1000 mg/day on days 2–6), clopidogrel (300 mg on day 1, and 75 mg/day from day 2), and aspirin (300 mg on day 1, and 100 mg/day from day 2). The attacks subsequently stopped. Catheter angiography showed stenosis of the left ICA from the proximal to distal portion and CBN (Fig. 4a, b). High intensity lesion on T1 W1 in the distal portion of the left ICA was observed (Additional file 2: Fig. S2B). MMV associated with GD was diagnosed. She refused any surgery. Methimazole (15 mg/day) was started, but mild thyrotoxicity continued (Fig. 3).

**Fig. 5:**
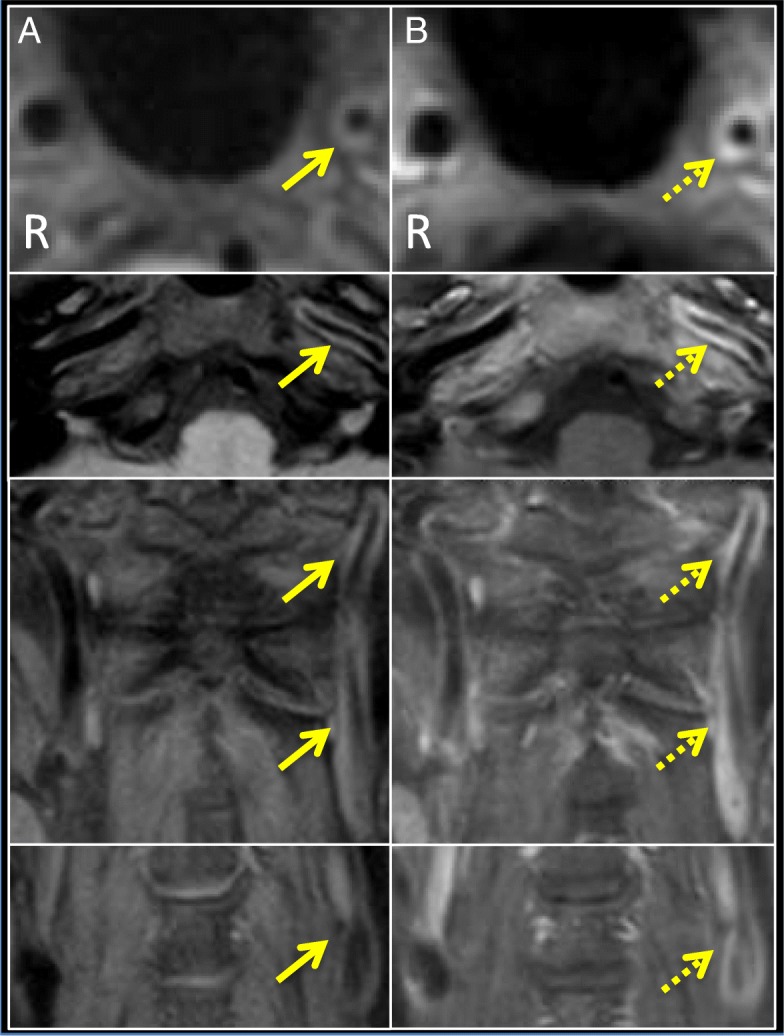
Radiological findings of vasculitis over the entire internal carotid artery. Six months after recurrence, 3D-T1WI (**a**) and CE 3D-T1WI (**b**) of the ICAs were performed (Upper 2 figures: axial images of the distal portion; Lower 2 figures: coronal images of the proximal portion in Figure **a**, **b**). **a** 3D-T1WI showed smooth, concentric wall thickening over the entire left ICA (arrow). **b** CE 3D-T1WI showed diffuse contrast enhancement on vessel walls (dashed arrow in Figure **b**) over the entire left ICA, suggesting vasculitis radiologically [23, 24]

We subsequently identified gradual progression in the left ICA and middle cerebral artery (MCA) on MRA (Fig. 1b-d) and 3D computed tomographic angiography. Finally, we found smooth, circumferential, concentric wall thickening with diffuse gadolinium enhancement of the left ICA from the proximal to distal portion on 3D-T1WI (Fig. 5a, b). We considered the possibility of vasculitis of medium-to-large vessels and administered prednisolone (PSL; 1 mg/kg/day) and methotrexate (MTX; 4 mg/week). Subsequently, thyroid function normalized (Fig. 3).

Six months later, PSL was reduced to 0.1 mg/kg/day, and MTX increased to 14 mg/week. Signal intensity in the left ICA and MCA increased on MRA. Catheter angiography showed development of net-like vessels, and incomplete occlusion of the ICA (Fig. 4c). By 18 months after recurrence, further improved flow in the left ICA and MCA on MRA was observed (Fig. 1e), but the vessel wall of the left ICA from the proximal to the distal portion still remained enhanced on CE 3D-T1WI in the same way as the previous one. Two years after recurrence, catheter angiography also showed improved blood flow in the left ACA, MCA and ICA, as well as mild improvement of stenosis of the terminal portion of the left ICA (Fig. 4d). We evaluated the polymorphism in c.14576 G > A (rs112735431) in the *RNF-213* gene, a susceptibility gene for Moyamoya disease, using genomic DNA samples from the patient. Genetic analysis of *RNF-213* showed wild type.

## Discussion and conclusions

### Review of the literature

We reported a patient with recurrent ischemic stroke and progressive stenosis of MMV associated with GD who had no obvious stenosis in the first stroke. We also reviewed previous reports with follow-up for over 4 months to clarify long-term relationships among recurrence of ischemic stroke, progression of MMV, thyrotoxicity and treatment. We conducted a Medline search for articles using the key words “cerebrovascular diseases”, “moyamoya”, “thyrotoxicity”, and “Graves’ disease”. To confirm long-term relationships, we selected case reports followed-up for over 4 months among these candidates [1, 2, 6-18], and excluded review articles and cases showing recurrence or repeated transient ischemic attacks within 3 months (Table 1), because we wanted to know whether treatment and control of GD over the long term was associated with recurrent ischemic stroke or progression of MMV. We also examined the presence of stenosis of ICAs, and champagne bottle neck sign (CBN) in all candidates [19].

**Table 1:** Reported cases of MMV associated with GD with follow-up for over 4 months

Case	Age (years)	Sex	Angio or MRA	Presentation	Thyroid condition at vascular accident	Treatment (thyroid)	Treatment (cerebrovascular disease)	Thyroid condition during follow-up	Neuro imaging	Recurrence	Thyroid condition at recurrence	Outcome	Follow-up period (months)	Reference		
1	29	F	Bilateral distal ICA stenosis	TIA	Thyrotoxic	ATM/PE	Aspirin	Normal range	Improvement	none	−	Good	6	Yamashita	7	2005
2	26	F	Rt ICA stenosis	Ischemic stroke	Thyrotoxic	ATMATM	Antiplatelet therapy	Normal range	Improvement	none	-	Good	12	Nakamura	8	2014
3	45	F	Bilateral distal ICA occlusion	TIA	Thyrotoxic	PSL/radioactive iodine	Antiplatelet therapy	Normal range	Improvement	none	-	Good	4	Uftk	9	2004
4	47	F	Bilateral net-like vessels	TIA	Thyrotoxic	PSL/radioactive iodine	**Aspirin**	n.d.	n.d.	none	-	Good	10	BEATRIZ	10	1997
5	37	F	Bilateral distal ICA stenosis	Ischemic stroke	Thyrotoxic	PSL/ thyroidectomy	Antiplatelet therapy	Normal range	n.d.	none	-	Good	6	BEATRIZ	10	1997
6	19	F	Bilateral net-like vessels	Ischemic stroke	Thyrotoxic	n.d	STA-MCA bypass	n.d.	n.d.	none	-	Good	24	Ran	11	2009
7	21	M	Rt distal ICA stenosis	Ischemic stroke	Thyrotoxic	ATM	Aspirin	Normal range	n.d.	none	-	Lt minor leg weakness	11	Carlos	12	1998
8	46	F	Right net-like vessels	Ischemic stroke	Hypothyroidism	none	STA-MCA bypass	Hypothyroidism	n.d.	none	-	Good	24	Ohba	2	2011
9	23	F	Bilateral distal ICA stenosis	Ischemic stroke	Thyrotoxic	ATM	none	n.d.	No change	none	-	Good	13	Nakamura	13	2003
10	54	F	Bilateral distal ICA stenosis	Ischemic stroke	Subclinical thyrotoxic	ATM	none	n.d.	No change	none	-	Good	6	Nakamura	13	2003
11	19	F	Bilateral net-like vessels	Ischemic stroke	n.d (immediately after thyroidectomy)	Thyroidectomy	STA-MCA bypass	n.d.	n.d.	none	-	Good	60	Tokimura	14	2010
12	35	F	Rt distal ICA stenosis	Ischemic stroke	Thyrotoxic	ATM/thyroidectomy	Cloidogrel	Normal range	No change	none	-	Good	6	Gon	5	2017
13	19	F	Bilateral net-like vessels	Ischemic stroke	Thyrotoxic	ATM	none	Thyrotoxic	n.d.	none	-	Lt hemiparalysis	60	Kushima	15	1991
14	26	F	Bilateral net-like vessels (recurrence)	TIA	Thyrotoxic	ATM	none	n.d.	n.d.	Recurrence	Thyrotoxic	Rt hemiparalysis	50	Kushima	15	1991
15	23	F	Bilateral distal ICA occlusion	Ischemic stroke	Thyrotoxic	ATM	Aspirin	Thyrotoxic	Progression	Recurrence (TIA) → EDAS	Thyrotoxic	Good	22	Shaneela	1	2011
16	16	F	Bilateral net-like vessels	TIA	Thyrotoxic	ATM	n.d	Thyrotoxic	Progression	Recurrence (Ischemic stroke)→ STA-MCA bypass	Thyrotoxic	n.d.	72	Im	16	2005
17	22	F	Rt MCA occlusion	Ischemic stroke	Thyrotoxic	ATM	Heparin edaravone in acute phase	Thyrotoxic	Progression	none	-	Lt hemiparalysis	36	Ishigami	17	2014
18	42	F	Bilateral net-like vessels	Ischemic stroke	Thyrotoxic	ATM (poor compliance)	EDAS	n.d.	n.d.	Recurrence	Thyrotoxic	Death	12	Ku	18	2015
19	15	F	Bilateral distal ICA stenosis	Non-automatic movement	Thyrotoxic	ATM	n.d	n.d.	Progression	Recurrence	Thyrotoxic	n.d	72	Ni	19	2014
20	37	F	No obvious stenosis	Ischemic stroke	Thyrotoxic	ATM (potassium iodine)	Warfarin	Thyrotoxic	Progression	Recurrence (TIA)	Thyrotoxic	Rt mild hemiparalysis	5	Our case		2017

*F* female, *M* male, *TIA* transient ischemic attack, *n.d.* not described, *Rt* right, *Lt* left, *ICA* internal carotid artery, *MCA* middle cerebral artery, *ATM* anti-thyroid medication (here, we define methimazole, propylthiouracil, or potassium iodine as ATM), *PSL* prednisolone, *PE* plasma exchange, *STA-MCA bypass* superficial temporal artery-middle cerebral artery bypass, *EDAS* encephoduroarteriosynangiosis

### Patients

The 20 patients included, 19 female and 1 male, with a mean age of 30.1 years (range, 15–54 years). The majority of the patients were female. Mean duration of follow-up was 22.3 months (range, 4–72 months).

### Thyrotoxicity and cerebrovascular disease in the first episodes

Eighteen patients (90%) showed neurologic symptoms during thyrotoxicity. One patient (5%) presented with subclinical thyrotoxicity and one patient (5%) was in a hypothyroid state.

### Treatment

For GD, 14 patients (70%) were treated with anti-thyroid medication (ATM), and 10 of these patients (50%) with ATM alone. Two patients (10%) were treated with only thyroidectomy, 2 patients (10%) with PSL and radioactive iodine therapy (RIT), and 1 patient (5%) with PSL and thyroidectomy. For MMV, 8 patients (40%) were treated with antiplatelet therapy, 1 patient (5%) with anticoagulant, and 4 patients (20%) with neurovascular surgery. There was no report of patients with no treatment.

### Outcomes—Recurrence and progression

Six patients (30%) experienced recurrence, and 5 patients (25%) showed progression. All of them showed thyrotoxic states at recurrence or progression and were treated with ATM alone for GD. One patient died of recurrence [17]. In contrast, among 5 patients (25%) treated with thyroidectomy or RIT, no patient experienced recurrence or progression. Three patients (15%) in a euthyroid state showed improved flow in the cerebrovascular arteries on MRA.

### Radiographic features

Based on our investigation of previous reports in the literature, our case represents the first description of ischemic stroke with no obvious stenosis of the cerebral arteries. Images of the proximaI ICA were available in 7 cases [1, 5, 12, 14, 20–22]. CBN was found in all cases.

The clinical course of our case is illustrated in Fig. 1. Thyroid function and titers of anti-TPO Ab and TRAb seemed to be associated with progression of MMV and occurrence of ischemic stroke. We found smooth, circumferential, concentric wall thickening with diffuse gadolinium enhancement over the entire left ICA, which is the radiological finding of vasculitis [23, 24]. Improved flow in the left ICA and MCA on MRA was observed after administration of PSL and MTX, and in the euthyroid state. The result of genetic analysis of *RNF-213* also suggests a different etiology of Moyamoya disease associated with c.14576 G > A variant in the *RNF-213* gene.

We noticed similarities between our case and Takayasu arteritis, including the involvement of a young female, findings of medium-to-large vessels on CE MR imaging, progression of stenosis in medium-to-large vessels, and elevated TAT [25, 26]. CRP and ESR can be negative in Takayasu arteritis [26]. In the first episode of our case, a hemodynamic etiology appeared unlikely as the cause of ischemic stroke, because stenosis of the left ICA was not obvious. It has been reported that although little, if any, endothelial change is evident, thrombus formation by vasculitis leads to ischemic stroke in Takayasu arteritis [25]. Thus, the same mechanism can be considered in our case. In the second episode, high intensity lesion on T1 W1 in the distal portion of the left ICA was observed (Additional file 2: Figure S2B). Dissection might be considered as the etiology of this lesion, because dissection shows eccentric wall thickening with T1 bright wall components representing intramural hematoma [24]. Moreover, vasculitis can cause aortic dissection, for example, Takayasu arteritis. T-cell-mediated immunity was reported to play important roles in Takayasu arteritis, GD and MMV [26–28]. Immunologic changes related to GD and MMV may have a common pathogenic link involving T-cell dysregulation [28]. The association between MMV and GD in our case and our review of the literature support this hypothesis.

CBN may be a characteristic feature of this disease and may be caused by vasculitis from the proximal ICA. CBN means a rapid, sharp reduction in internal diameter at the proximal ICA, which is observed in some patients with moyamoya disease [6]. In our case, the difference between thickness of the wall of the left and right ICA seemed to become clear gradually (Fig. 5a, b Additional file 2: Figure S2A, B), possibly due to vasculitis. This seemed to contribute to the formation of stenosis of the entire ICA and CBN in our case (Additional file 3: Figure S3). In other cases of MMV associated with GD or moyamoya disease with CBN, the same process might occur. Since the vessel lumen is narrower in the distal than in the proximal portion, occlusion may occur in the distal portion.

Treatment for MMV associated with GD by ATM alone may be risky in terms of recurrence. So far, the treatments, especially without surgery, have not been sufficiently studied. For GD, ATM, RIT, thyroidectomy, PSL and PE were used in previous reports. For MMV, treatments should reportedly be performed the same way as for moyamoya disease through antiplatelet agents and bypass surgery [29]. The similarities with Takayasu arteritis encouraged us to treat our patient with PSL and MTX as treatments for vasculitis, although, to the best of our knowledge, there has been no report of such treatment for this type of patient. Improved flow of the left ICA and MCA on MRA was observed after administration of PSL and MTX, and in the euthyroid state. On the other hand, such a favorable result, the improved flow, can not occur in Moyamoya disease, in which MCA disappears as the next stage in the Suzuki stage [29]. Considering a retrospective study in which progression advanced even when GD was controlled [4], the improvement on MRA in our case might have been due to the treatment of vasculitis. IVMP in the acute phase in the second episode might have worked for both thyrotoxicity and vasculitis. According to our review, recurrence or progression was found in patients treated with ATM alone, while recurrence or progression was not found with RIT or thyroidectomy. This seems to be due to the higher recurrence rate (range, 50–67%) for GD under the usual treatment with ATM compared to 15% with RIT and 10% with thyroidectomy [30]. Notably, 1 patient treated with ATM alone died following recurrence [17]. Since RIT was reported to increase the risk of cerebrovascular events [31], thyroidectomy may be reasonable choice to reduce the risk of recurrence or probable progression.

Our review was limited by the retrospective nature of the data collection. For example, duration of follow-up tended to be longer in recurrent cases. Despite these limitations, the overall review provides important lessons on recurrence.

## Conclusions

This report suggests the possibility of vasculitis of medium-to-large vessels in MMV associated with GD. This is the first case where cerebral infarction occurred during thyrotoxicity without obvious stenosis of ICAs. We treated the patient as having vasculitis of medium-to-large vessels with IVMP in the acute phase and with PSL and MTX in the chronic phase. Subsequently, improved flow in the cerebrovascular arteries on MRA was observed in the euthyroid state. Treatment with ATM alone seems to be risky in terms of recurrence. Adequate management of GD and possible vasculitis may be important for preventing recurrence and progression.

## Additional files

Additional file 1: **Figure S1.** MR imaging of cerebral infarction in the first episode. Axial diffusion-weighted images showed infarcts in the left cerebral hemisphere. (PDF 202 kb)

Additional file 2: **Figure S2.** Coronal 3D-T1WI of ICA in the first and the second episode. (A): Image in the first episode. The vessel walls might be thicker over the left entire ICA compared to the right, but it was not clear. (PDF 322 kb) (B): Image in the second episode. Stenosis became severe, especially in the distal portion of the left ICA. High intensity lesion on T1 W1 in the distal portion of the left ICA (arrow) was observed. Dissection might be considered as the etiology of this lesion, because dissection shows eccentric wall thickening with T1 bright wall components representing intramural hematoma [24]. Moreover, vasculitis can cause aortic dissection, for example, Takayasu arteritis.

Additional file 3: **Figure S3.** Hypothetical process for the formation of the CBN and stenosis over the entire ICA and its relationship to MMV. We hypothesize that vessel wall thickness by vasculitis contributes to the formation of the CBN and stenosis of the entire left ICA on MRA. This is because they became more evident in the second episode than in the first episode in our case. Since the vessel lumen is narrower in the distal than in the proximal portion, occlusion may occur in the distal portion. (PDF 204 kb)

## Abbreviations

3D: three-dimensional
anti-TPO Ab: anti-thyroid peroxidase antibody
ATM: anti-thyroid medication
CBN: champagne bottle neck sign
CE: contrast-enhanced
CRP: C-reactive protein
DWI: diffusion-weighted imaging
ESR: erythrocyte sedimentation rate
GD: Graves’ disease
ICA: internal carotid artery
IVMP: intravenous methylprednisolone
MCA: middle cerebral artery
MMV: moyamoya vasculopathy
MRA: magnetic resonance angiography
MTX: methotrexate
PSL: prednisolone
RIT: radioactive iodine therapy
T1WI: T1-weighted imaging
TAT: thrombin-antithrombin complex
TRAb: TSH receptor antibody
TSH: thyroid stimulating hormone
