# Supplementary figures and images for: Progressive stenosis and radiological findings of vasculitis over the entire internal carotid artery in moyamoya vasculopathy associated with graves’ disease: a case report and review of the literature
Source: BMC Neurol. 2019 Mar 2;19:34. doi: 10.1186/s12883-019-1262-1 (PMC6397453; doi:10.1186/s12883-019-1262-1)

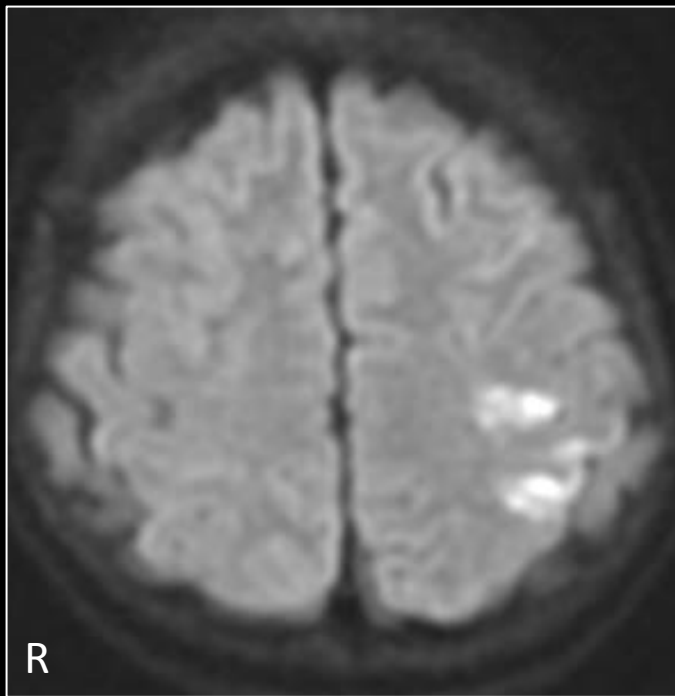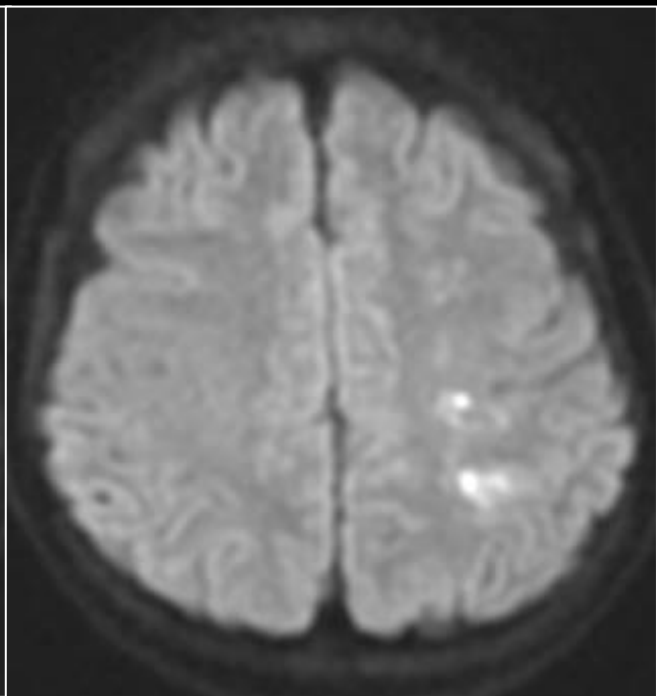

Supplement: Supplementary file 1 — Figure S1. MR imaging of cerebral infarction in the first episode. Axial diffusion-weighted images showed infarcts in the left cerebral hemisphere. (PDF 202 kb) [file 12883_2019_1262_MOESM1_ESM.pdf]

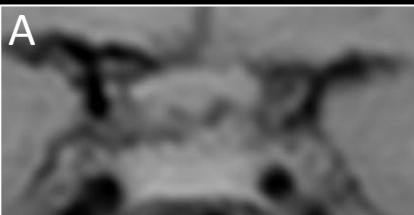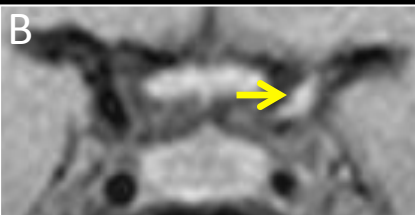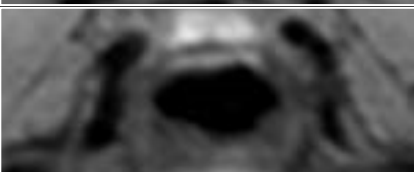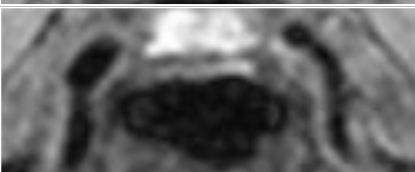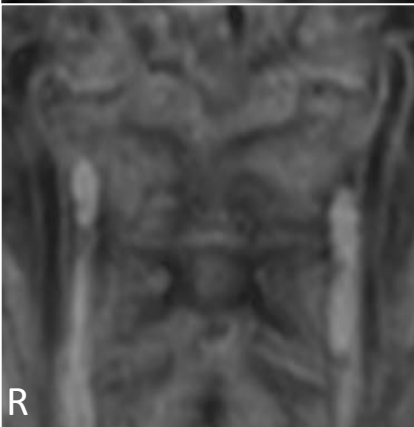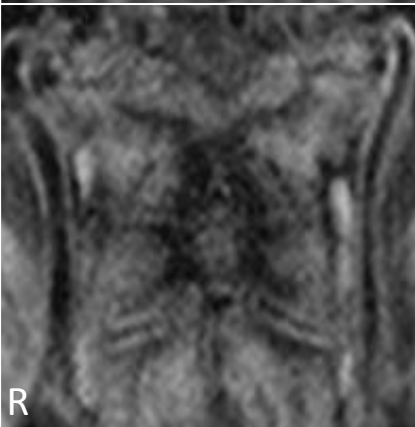

Supplement: Supplementary file 2 — Figure S2. Coronal 3D-T1WI of ICA in the first and the second episode. (A): Image in the first episode. The vessel walls might be thicker over the left entire ICA compared to the right, but it was not clear. (PDF 322 kb) (B): Image in the second episode. Stenosis became severe, especially in the distal portion of the left ICA. High intensity lesion on T1 W1 in the distal portion of the left ICA (arrow) was observed. Dissection might be considered as the etiology of this lesion, because dissection shows eccentric wall thickening with T1 bright wall components representing intramural hematoma [24]. Moreover, vasculitis can cause aortic dissection, for example, Takayasu arteritis. [file 12883_2019_1262_MOESM2_ESM.pdf]

First episode

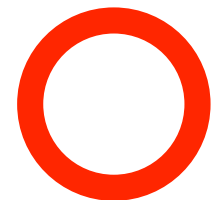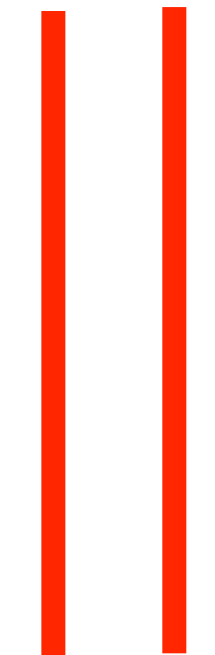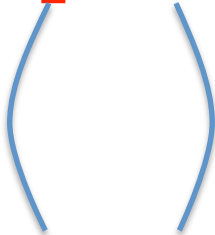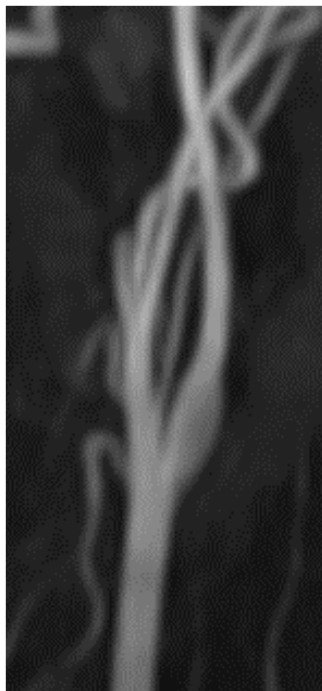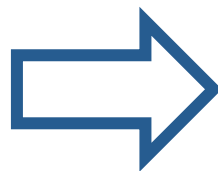

Second episode  
During progression

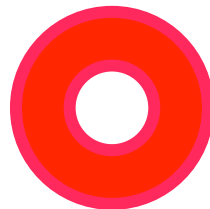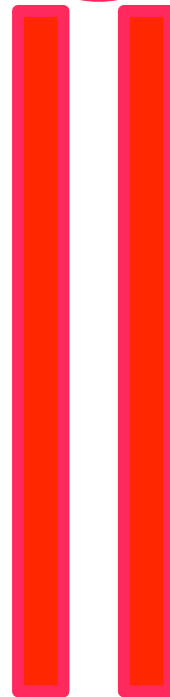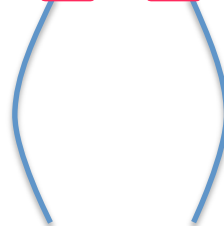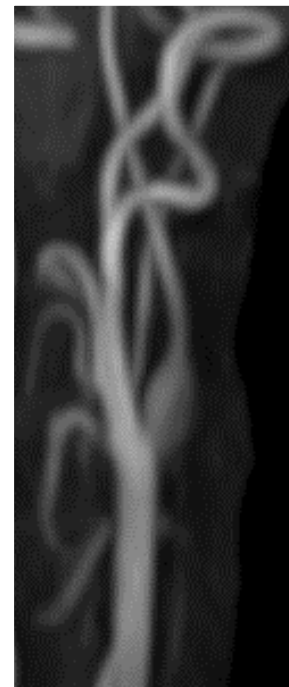

Supplement: Supplementary file 3 — Figure S3. Hypothetical process for the formation of the CBN and stenosis over the entire ICA and its relationship to MMV. We hypothesize that vessel wall thickness by vasculitis contributes to the formation of the CBN and stenosis of the entire left ICA on MRA. This is because they became more evident in the second episode than in the first episode in our case. Since the vessel lumen is narrower in the distal than in the proximal portion, occlusion may occur in the distal portion. (PDF 204 kb) [file 12883_2019_1262_MOESM3_ESM.pdf]
